# Light-driven phenotypic plasticity in the depth-generalist coral, *Pavona varians*

**DOI:** 10.1371/journal.pone.0326069

**Published:** 2025-07-01

**Authors:** Claire J. Lewis, Shayle B. Matsuda, Tayler L. Sale, Caitlyn Genovese, Chelsea S. Wolke, Norton Chan, Stephen Ranson, Jake M. Ferguson, Amy L. Moran, David A. Gulko, Peter B. Marko

**Affiliations:** 1 School of Life Sciences, University of Hawaiʻi at Mānoa, Honolulu, Hawai‘i, United States of America; 2 Hawaiʻi Institute of Marine Biology, Kāneʻohe, Hawai‘i, United States of America; 3 Conservation Research Department, John G Shedd Aquarium, Chicago, Illinois, United States of America; 4 USFWS Pacific Islands Fish and Wildlife Office, Honolulu, Hawai‘i, United States of America; 5 Division of Aquatic Resources, Hawaiʻi Department of Land and Natural Resources, Ānuenue Fisheries Research Center, Honolulu, Hawai‘i, United States of America; 6 Hawaiʻi Coral Reef Initiative, Social Science Research Institute, University of Hawaiʻi at Mānoa, Honolulu, Hawai‘i, United States of America; 7 Kerckhoff Marine Laboratory, California Institute of Technology, Pasadena, California, United States of America; 8 Department of Biology, University of Kentucky, Lexington, Kentucky, United States of America; University of the Ryukyus, JAPAN

## Abstract

Climate change is causing shifts in the spatial distribution of species and a reshuffling of the composition of multiple community types. On coral reefs, deep water can act as both refuges and refugia for corals from the combined negative effects of heat and light stress. Phenotypically plastic generalists that can tolerate both low and high light environments could be disproportionately important on future reefs, persisting in refugia and colonizing vacant shallow reefs. We performed a common garden experiment to investigate the effect of light on three different wild-collected genotypes of the abundant, depth-generalist coral *Pavona varians*. We measured the growth response and reaction norms of six other morphological and functional traits in full sunlight, 75%, and 90% shade. We also modeled the combined effects of light and temperature on growth. *P. varians* had positive growth in all three treatments, but increased both skeletal mass and 2-D colony footprint most in 90% shade, with a higher density of corallites, and a less rugose skeleton that may enhance light capture. Areas of the colony corresponding to new growth had greater fluorescence of Symbiodiniaceae communities in the darkest treatment. Light did not alter the functional lipid ratio, nor did communities of Symbiodiniaceae vary with light treatments. The model revealed additively negative, but not synergistic, effects of light and temperature on growth. This additively negative relationship in the model is consistent with the hypothesis that reductions in bleaching at depth could be the product of reduced light stress at depth rather than reduced temperature stress. Light-associated plasticity likely allows *P.varians* to live in a wide variety of habitats and across a broad depth gradient. In reduced light conditions, this species may mitigate some of the negative effects of bleaching temperatures on growth. We predict that *P. varians* is likely one of a minority of species that may benefit from deep reef refugia.

## Introduction

Phenotypic plasticity allows multiple phenotypes to be expressed by a single genotype in response to different environments [1–3]. Greater phenotypic plasticity is often associated with species that are predicted to do well in a changing climate, such as successful invaders [4], species with large distribution ranges [5], and those able to persist in heterogeneous environments [6–8]. Phenotypic plasticity may allow species to tolerate rapidly changing climates in two ways: sessile or modular species may alter their phenotypes in place over their lifespans, and for species with dispersive life-history stages, plasticity may facilitate range-shifts or extensions into less stressful habitats [9–12]. Therefore, plasticity of foundational autotrophs, organisms which contribute to the ecosystem engineering of terrestrial and marine forests, such as some corals and plants, may be particularly important to understand as the climate changes [13–16].

Climate change might cause coral species to shift their distributions from increasingly stressful shallow-water habitats on tropical reefs to deeper water, potentially via larval dispersal combined with plasticity. The Deep Reef Refugia Hypothesis (DRRH) proposes that mesophotic reefs, at depths of 30 m or more, may act as refugia for depth generalists locally extirpated from shallow reefs by a multitude of stressors such as storms, pollution, disease, and overfishing [17, 18, 19], but see [20]. Climate change is causing oceans to warm, leading to more frequent heat waves, bleaching events, and mass mortality on coral reefs [21]. However, coral bleaching, a breakdown in the symbiotic relationship between coral hosts and their intracellular zooxanthellae (Family: Symbiodiniaceae), is often caused by the combined stress of high temperature and light [22, 23], both of which decline with depth [19]. Accordingly, bleaching severity is often reduced under low light, such as at depth [24–27] and during periods of high cloud cover [28, 29] and increased turbidity [30–33]. Although the low light which characterizes the mesophotic may generally slow coral growth [34], all else being equal, reduced irradiance of deep reefs likely reduces symbiont photosystem stress during high temperature events [35]. Other stressors and their interactions are likely important [36, 37], but the extent to which temperature and light additively or synergistically negatively affect coral growth is not totally clear, and may not be consistent across species [38, 39].

The realized impact of deep reefs as ecological refuges and evolutionary refugia is limited by the disparity in coral community composition between deep and shallow reefs. Based on the distribution of their abundances, most corals are likely depth specialists: the dominant reef builders on both mesophotic and shallow reefs tend to be species with specialized, inflexible morphologies, limiting ecological exchangeability across depth gradients [19, 40, 20, 41, 42]. In contrast, the handful of depth generalist species capable of persisting at demographically-significant densities from shallow to deep reefs, may do so via light-associated phenotypic plasticity [41]. These plastic generalists may be particularly important in a warming ocean as they are able to tolerate the broad range of light conditions needed to use deep reefs as refuges [41, 43]. Further complicating this issue is the ongoing difficulty of accurately identifying coral species, particularly at depth where survey efforts are reduced and morphology may be altered by the changed abiotic conditions [44, 45].

**Fig 1:**
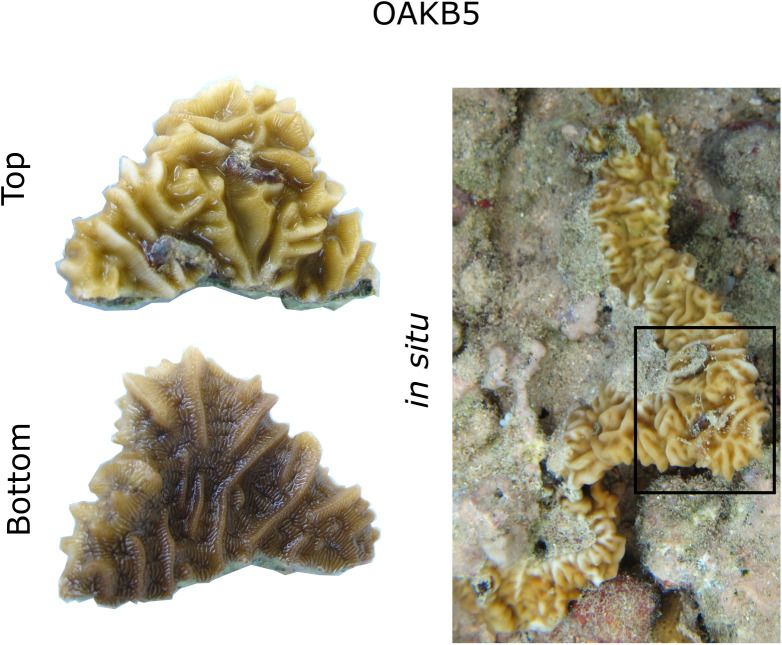
Colony OAKB5 in situ, and top and bottom of collected fragment. Observation of intracolony variation in gross morphology between the exposed top and shaded bottom of a *P. varians* colony, suggesting light-associated phenotypic plasticity.

The agaricid coral *Pavona varians* [46] is considered a depth generalist [41, 42], found at depths from 0.5 to > 50 meters [47]. One of the most common species in the Indo-Pacific [48, 49], ranging from the Red Sea to the Eastern Pacific [50], *P. varians* is aptly named, exhibiting high variation in both microskeletal structure and gross colony morphology [51]. Unrecognized cryptic diversity may be responsible for some morphological variation in this nominal species [50]; however, depth [52], wave energy [51], and latitude [50] may also explain inter-colony morphological variability of this species. Naturally occurring intracolony variation suggests *P. varians* is phenotypically plastic with respect to light because individual colonies can be more rugose on upward-facing surfaces than on downward-facing surfaces (Fig 1).

To investigate the extent of light-driven phenotypic plasticity in *P. varians*, we conducted a common garden experiment in outdoor aquaria using three different field-collected colonies that were genetically distinct. We manipulated light over a ~ six-month period and measured the effects on: lateral growth rate, calcification, corallite density, skeletal rugosity, coral tissue thickness, lipid content and composition, and the density and genetic identity of the community of Symbiodiniaceae. All of these factors have been used as metrics of coral physiological condition or are known to be relevant to their functioning in the ecosystem [53–55]. We were particularly interested in the effect of light on skeleton rugosity, because more and taller ridges may have functional importance for this species by modulating the internal light environment experienced by symbionts. Assessing plasticity of growth rate (measured here as lateral growth, calcification, and rugosity), is particularly important because growth is likely a key means of tolerating a rapidly changing climate [15] and provides specific insight into how corals respond to changing environmental conditions and how they allocate energy to different physiological processes [56,57]. Growth rate is also the primary way that corals are classified into functional groups [53], which are used to make predictions about climate-driven changes in community structure on coral reefs [57]. Although coral growth depends fundamentally on environmental conditions [58], the impacts of spatial variation in light, temperature, and other environmental drivers are rarely considered in trait-based classifications, largely because of a lack of experimental data [40].

In addition to fragmenting and growing colonies under manipulated natural lighting, we then took advantage of natural seasonal variation in light and temperature over the experiment to model and statistically separate the effects of light and temperature on growth. Our approach allowed us to assess the proposed synergistic effects of heat and light on coral growth [e.g., 37], and understand the physiological correlates underlying the potential benefits of deep reefs for generalist species in a warming ocean.

## Methods

### Coral collection

The three colonies of *P. varians* included in the common-garden experiment were collected by hammer and chisel with snorkel or SCUBA by Hawaiʻi Division of Aquatic Resources (DAR) personnel, and therefore no permit was required as they are the regulating agency for coral collection in Hawaiʻi. Colony 1 was collected 1m deep in (Winter 2015) from Ke’ehi Boat Harbor, O’ahu (21 18’ 43.4” N, 157 53’ 22.4” W) while colonies 2 and 3 were collected 3m deep in (Spring 2017) at Sand Island State Recreation Area, O’ahu (21 17’55.76”N, 157 52’ 24.07” W). These colonies were transported <10 minutes to the Hawaiʻi Division of Aquatic Resources’ Ānuenue Fisheries Research Center’s Coral Restoration Nursery, on Sand Island. All colonies were kept in running seawater under two layers of shade cloth until the experiment began.

A fourth colony, which was not used in the common-garden experiment, “Colony OAKB5”, was collected from 1 m deep (Summer 2017) from Kāne’ohe Bay (21°27’10.0”N, 157°48’03.4”W, Hawai’i DAR: Special Activities Permit 2016−57). This colony (shown in the field in Fig 1) was used to represent an *in situ* colony where large light differences across the colony may be effecting the symbiont community. PCR and sequencing of mitochondrial DNA confirmed all four colonies as the same phylogenetic group of *P. varians* recovered by [47](see SM for details, S1 Fig). The three colonies used in the long-term experiment were genetically distinct from each other at the same locus, so we considered them to be biological replicates.

### Experimental design

To test the effect of light on our multiple metrics, a ~ 6-month experiment was conducted at the Hawaiʻi Division of Aquatic Resources Āʻnuenue Coral Restoration Nursery, Sand Island (21°18’14.6“N 157°52’16.1”W) on the island of Oʻahu, Hawaiʻi. The experiment was conducted outdoors in two 3.9 m diameter flow-through tanks, supplied by running, unfiltered sea water. We used natural running seawater rather than maintaining a fixed tank temperature, allowing us to incorporate natural thermal variation into our statistical models over the six-month experiment. Each tank was lit via natural sunlight and divided into six equally-sized triangular segments to which three light treatments were applied using zero, two, and four layers of 50% shade cloth, corresponding to 0, 75, 90% shade respectively (S2 Fig). These treatments were randomly applied in duplicate in each tank, such that each treatment was replicated four times across the two tanks. The difference in irradiance between 0, 75% and 90% shade, as measured in our experiment by HOBO loggers, was roughly equivalent to descending 33 m and 56 m respectively in the clear, tropical waters of Hawaiʻi [54]. We recognize, however, that the light spectrum is attenuated unevenly with increasing depth, thus our shade cloth treatments would not capture the complete effects of depth on light.

On June of 2017, each of the three genetically distinct *P. varians* parent colonies were cut into twelve 1 cm^2^ fragments using a diamond bandsaw, and each fragment was glued to individual 5 cm^2^ unglazed travertine tiles using IC-GEL ethyl cyanoacrylate gel glue (BSI Inc.) and left to recover for two weeks under two layers of 50% shade cloth in running sea water. The experiment was started on June 19^th^, 2017; one fragment from each genotype was randomly placed in each of the twelve treatments/replicates (three light treatments, each separately replicated four times) and placed on a plastic tray suspended 10 cm below the surface in the middle of the treatment area. Because of constraints imposed by the design of the aquarium facility, the light treatment replicates were nested within the two tanks; therefore, tank was included as a fixed factor in the statistical analysis (see below) (S2 Fig). Each replicate included a HOBO pendant light (lux) and temperature (°C) logger, recording both variables every ten minutes for the duration of the experiment. For summary statistics, lux was converted to watts m^-2^ using a conversion factor of 0.008196721 [59]. The unoccupied portions of tiles were cleaned weekly using a toothbrush to maintain a uniform environment across treatments with respect to sediment and fouling communities.

### Data collection

At the beginning of the experiment and monthly thereafter, tiles (and attached coral fragments) were buoyantly weighed (following the protocol described in [60]) to the nearest 0.025 g using an electronic balance (Mettler Toledo MS8025), and calibrated photographs were taken from directly above the two-dimensional (2D) footprint of each coral.

At the end of the experiment (data was collected over a two week period from November 17^th^ 2017 to December 1^st^ 2017), each fragment was weighed to estimate calcification, photographed for linear growth measurements and to estimate rugosity, and then each fragment was divided into several smaller subfragments of approximately 1 x 1 cm size and used for laser scanning confocal microscopy (symbiont density and tissue thickness), ash-free dry weight and proximal lipid content and composition, and genetic identification of symbiont community. Fragments for lipid content were immediately frozen on dry ice before being transported to a −80°C freezer. Samples of the fragments for fluorescence and tissue thickness measurements were immediately preserved in Z-fix (Anatech Ltd, Battle Creek, MI) and stored at 4°C in the dark.

Hereafter we will refer to the three parent corals as colonies or genotypes, equivalent to genets, and fragments associated with each genotype will be referred to as fragments, equivalent to ramets [61]. For the remainder of the paper, the fragment originally glued to the tile (including vertical growth during the experiment) will be referred to as the “original fragment”. New growth around this original fragment will be referred to as “new lateral growth”.

### Lateral growth, weight, and corallite density

Change in skeletal weight was measured by subtracting the initial buoyant weight of the fragment + tile from the final buoyant weight at the end of the experiment (151 d), and dividing by the total number of days in treatment to get a daily rate. This method assumes that the density of organic coral tissue is the same as water, so the underwater weight represents the weight of the calcified skeleton and any change over time is due to an increase (or decrease) in the amount of calcified skeleton [60]. Change in the 2D surface area of living coral tissue was measured from top-down photographs with a ruler for scale (Canon EOS 6D, 135 mm lens). 2D surface areas were measured in Adobe Photoshop (v. 19) to calculate area in cm^2^. To calculate 2D colony growth, area measurements from the first and last time point were log transformed to account for the circular nature of growth and transformed into a daily rate. For the three fragments which had negative growth (all in the 0% shade treatment), the rate was fixed at zero, to ensure no exaggeration of growth differences in the 75% and 90% shade treatments.

Corallite density was calculated in areas of new lateral growth because of the difficulty in counting corallites between ridges on the original fragments. Corallites were counted in ImageJ (v.1.8.0_77). The number of corallites were then divided by the area of new growth, determined by subtracting the area of the original fragment subtracted from the final area measurement.

### 3D photogrammetry and fragment rugosity

We characterized colony rugosity with photogrammetry to assess the extent of ridge formation in the fragments. Each colony was photographed 35–50 times (depending on fragment size) in two concentric circles, with a ruler for scale and reflective disk for reference. 3D photomodels were created using Agisoft Photoscan Professional (v.1.2.6) (now Agisoft Metashape) using default settings, [62,63,64]. 3D surface area was measured in GeoMagic Control X 64 (v.2018.0.1). For three fragments which had negative growth all from the 0% shade treatment, photomodels were not created because in the absence of new growth, we would not be able to calculate the effect of light morphological changes of colony rugosity.

To calculate fragment rugosity, the 2D surface area (i.e., the 2D fragment footprint calculated earlier) was divided by the 3D surface area, and the difference scaled to the 2D surface area. The 3D surface area was always greater than the 2D, with increases in their ratio correlating with higher rugosity.

### Coral tissue thickness and symbiont densities

We used Laser Scanning Confocal Microscopy to measure the intensity and depth of fluorescence of Symbiodinaceae and coral host tissue in each fragment. These two factors can be used to estimate symbiont density and tissue thickness, respectively, which can in turn be used to infer coral health, see [55]. Two pieces (<1 cm^2^ each) of each coral, one from the original fragment and one from new lateral growth, were fixed in 1:4 zinc-buffered formalin (Z-Fix Concentrate, Anatech, Ltd) in 1 μm filtered seawater, and stored at 4°C in darkness. Samples were imaged using a Zeiss LSCM 710 and Zen Black software (2011 v14.0.16.201), with an excitation wavelength of 405 nm with 2.5x magnification. For tissue thickness, 3D models were sliced vertically at three randomly generated points across the x-axis; the mean of five depth measurements at each cross-section were averaged to generate a tissue depth score per sample. For symbiont densities, 3D models were generated (threshold 1.12 ± 0.2) and collapsed into 2D Maximum Intensity Projection maps. The average Symbiodiniaceae fluorescence score of three non-overlapping uniform ROI’s (n = 3/fragment) was then calibrated to a percent relative intensity score using the InSpeck Microscope Image Intensity Calibration Red Kit (Molecular Probes, Sigma-Aldrich, St. Louis, MO).

### Lipid content, dry weight and ash-free dry weight

Two sub-fragments (0.8–2 g) from each of the new lateral and original growth of each *P. varians* sample were placed in a mortar and pestle, crushed, and mixed into a homogeneous slurry by adding 1 ml DPEC water for every 1 g of coral [65,66]. Dry weight (DW) was measured by taking two 100-ul aliquots of the coral slurry and placing it in separate pre-ashed pans that were dried to a constant weight at 60°C for 3 h. Dried sub-fragments were then ashed at 450°C in a muffle furnace to a constant weight (AW). Ash-free dry weight (AFDW) for each sample was calculated as the difference between DW and AW. The mean of the two aliquots was used to estimate DW, AW, and AFDW.

### Lipid analysis

To quantify lipid class abundance and composition, whole coral slurries were prepared as above and lipids were extracted and quantified as in [66]. Briefly, lipids were extracted from two replicate 100-µl aliquots of coral slurry from each of 29 sub-fragments. Three fragments were excluded from the lipid analysis because the fragment was too small to provide enough material for all of the destructive analyses, and we chose to prioritize the fluorescence measurements due to stronger a priori expectations, please see data for which fragments were analyzed. Lipids were extracted in 2:1 methanol/chloroform for 30 min at −20°C, washed in 1:1 chloroform/water dried under a N_2_ gas stream, and resuspended in 30 µl chloroform. Two 1 µl subsamples of each of the two aliquots were spotted on quartz-impregnated chromatographic rods and run through a two-step development process [66,67]. Total lipid mass fraction was calculated as the mass of the summed lipid classes divided by the total AFDW of the sub-fragment [66]. The functional lipid ratio (FLR) [68], the ratio of storage to structural lipids, for each sample was calculated by summing the mass of all non-polar (storage) lipids and dividing by the mass of all polar (structural) lipids. The FLR can be an indicator of growth rate, photosynthesis and respiration rates, or reproductive condition [69–71].

### Photosynthetic symbiont community structure

Symbiodiniaceae communities were characterized from DNA extracted from the initial coral slurry generated during the lipid extraction using a Qiagen DNeasy kit. We assessed symbiont community for twelve experimental fragments: two from the 0% and 90% shade treatments for each of the three genotypes. Due to resource constraints we decided to look at only the two most different light treatments where light-driven differences in symbiont communities were most likely to be. Libraries were also prepared for the top and bottom sections of a single colony showing phenotypic plasticity in the field (OAKB5, not included in the light experiment). The rRNA ITS2 region was amplified with primers ITSDINO-F [72] and ITS2REV2 [73] that included adapters (Illumina, USA) added to the 5’ end of each primer. Amplifications were conducted in 13 μl volumes consisting of 6.3 μl MyTaq 2x (Bioline, USA), 0.3 μl of each 10 μM primer, 0.65 μL 20 mg/mL BSA, 4.65 μl Ultrapure distilled water (Life Technologies), and 0.5 μl DNA. Thermal cycling consisted of an initial denaturation step of five min at 95°C, followed by 35 cycles of 30 s at 95°C, 30 s at 53°C, and 45 s at 72°C, with a final extension of 10 min at 72°C. Nextera XT (Illumina, USA) index adapters were added to each library followed by purification with Agencourt©Ampure XP beads. Amplicons (including negative controls) were then (pair-end, 300 bp) sequenced on an Illumina MiSeq platform at the ASGPB. Sequences were analyzed using SymPortal [74] to generate ITS2 profiles: a holisitic assessment of the Symbiodiniaceae community.

### Statistical analysis of endpoint sampling metrics

All growth and tissue metrics from the end of the experiment were analyzed with linear mixed effects models using lme4 (v1.21) [75] and lmerTest (v3.1) [76] in R (v3.5.2). Treatment and genotype were fixed effects with an interaction term, with tank also as a fixed effect due to their being only two levels. Normality and homogeneity were confirmed using residual and q-q plots. Cook’s Distance was used to remove between 0–3 outliers from each response variable. For datasets with significant treatment or genotype effects, post hoc pairwise comparisons were performed using a Tukey HSD test, using emmeans package (v1.3.5.1) [77] in R.

### Statistical Analysis of the effect of light and temperature on lateral growth

We leveraged natural seasonal variation in environmental conditions to investigate the effects of light and temperature on growth measurements taken approximately every 30 d. We calculated average temperature and average peak light (between 10 am-3 pm) from data logged by HOBO loggers for each one-month time period between photographs (five in total). Lateral growth was used for shorter-term intervals because it responded more rapidly to the environment than buoyant weight, where tissue death may not result in skeletal loss for many months. Growth, temperature and light data for 36 tiles and from five time periods were included (180 data points); 3 data points were identified as statistical outliers due to likely logger malfunction and removed from further analysis, for a total of 177. Growth for each month was measured similar to section 2.4 areas the log difference in area. Light and temperature were both scaled to one to account for extreme differences in variation between the two metrics (e.g., 2,000–40,000 lux vs. 26–28°C).

Because we expected lateral growth to be influenced by the original size of the fragment (the growing edge increased as the fragments got larger), the previous month’s area was included in the model (LaggedArea). Further, the time periods were not equal so Days between measurements were included, as were Tank and Tile, as random effects. We explored increasingly complex models with additive and/or interactive effects between light, temperature, and genotype, and selected the model with the lowest AICc (Akaike Information Criterion with a small sample-size correction).

We compared the best Linear Mixed Effect models to a Generalized Additive Model with the same fixed effects to account for potential nonlinear responses in growth rates to predictors. Because AICc cannot directly compare models with and without random effects, we used cross-validation to assess each model’s mean-squared predictive error, the ability to predict data that has been held out of the fitting process. The predicted effects of light and temperature on growth under an LME model were visualized using sjPlot (v2.7.2), with distribution of input data overlain as a rug plot using ggplot2 (v3.2.1) [78] in R.

## Results

### Experimental conditions

Temperature and light varied seasonally over the 6-month duration of the experiment (S3 Fig). Peak light levels ranged from 309 to 585 watts m^-2^ (mean = 442 watts m^-2^), 72–123 watts m^-2^ (mean = 101 watts m^-2^), and 25–41 watts m^-2^ (mean = 34 watts m^-2^) in the 0, 75 and 90% shade treatment levels, respectively. Temperature ranged from 26.9 to 28.1°C (mean = 27.1°C), 26.6 to 27.8°C (mean = 26.9°C), and 26.6 to 27.7°C (mean = 26.9°C) in the 0, 75 and 90% shade treatments, respectively. Four fragments bleached in the first month of the experiment and suffered partial mortality. These fragments were in the 0% shade treatment and from Genotype 3; all were growing and had recovered pigmentation by the end of the experiment.

### Growth and skeletal morphology

**Fig 2:**
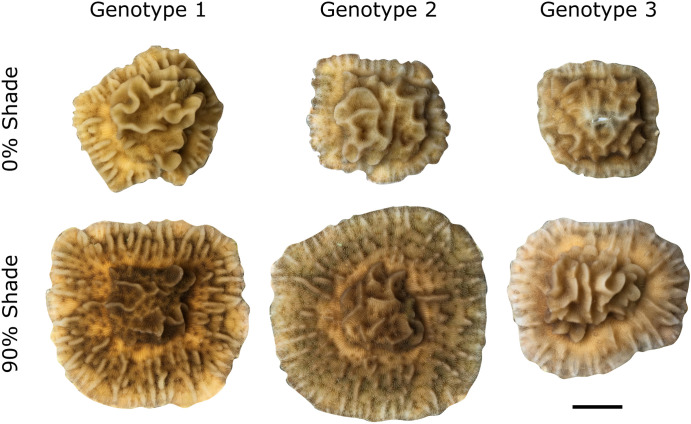
Photographs of example colonies from all three experimental genotypes from 0 and 90% shade treatments, scale bar 1 cm, at the conclusion of the 6 month experiment.

**Fig 3:**
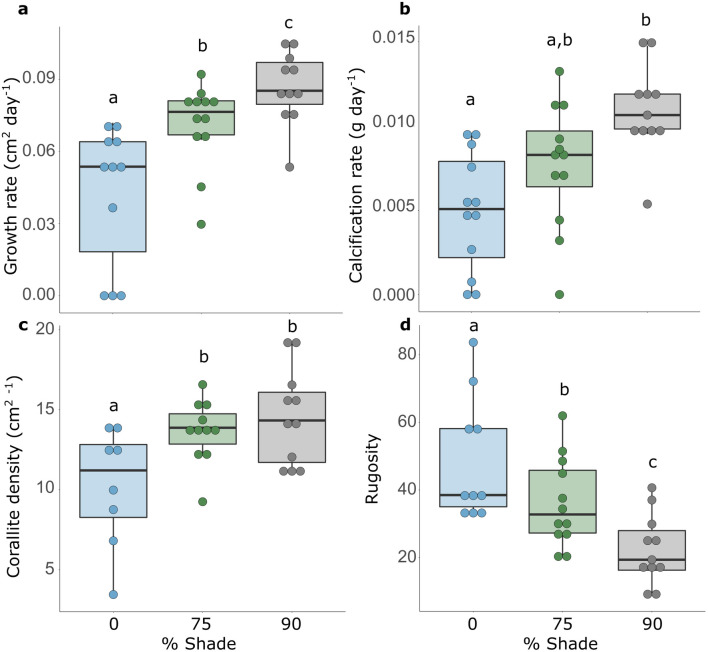
Growth and morphological metrics of *Pavona varians* across three light treatments: a) growth rate, b) calcification rate, c) corallite density on lateral growth, d) colony rugosity. Letters correspond to significant pairwise differences in post hoc analysis (p < 0.05).

There was an overall significant negative effect of light level on growth and growth-related traits. Calcification rate was significantly affected by light (LM: df = 2, F = 14.1, p = 0.00005), genotype (df = 2, F = 7.6, p = 0.0023), and tank (LM: df = 1, F = 4.6, p = 0.04) (Fig 2). Fragments deposited twice as much skeleton in the darkest treatment (mean = 0.011 g day^-1^ ± 0.0012) compared to full sun (mean = 0.005 g day^-1^ ± 0.001) (Fig 3a).

Lateral coral growth rate was also affected significantly by light (LM, light: df = 2, F = 37.9, p < 0.0001, genotype: df = 2, F = 28.73, p < 0.0001), and the interaction between light and genotype was also significant (light*genotype: df = 4, F = 3.2, p = 0.03). The effect of tank was not significant. Fragments grew more than twice as fast laterally in the 90% shade treatment (mean = 0.085 cm^2^ day^-1^ ± 0.00369) compared to the full sun treatment (mean = 0.039 cm^2^ day^-1^ ± 0.00369), and this effect was particularly strong for Genotype 3 (S4a Fig).

Fragments not only grew more in the darkest environment, but they also grew differently with respect to fragment morphology and organization. First, whole fragment rugosity was significantly affected by light (LM, light: df = 2, F = 22.8, p < 0.001) and genotype (genotype: df = 2, F = 21.9, p < 0.0001): the ratio of 3-dimensional to 2-dimensional surface area was twice as large in full sun (46.24 ± 2.95) as in the darkest treatment (21.4 ± 2.7) (Fig 3c). There was no significant interaction between light and genotype, nor was the single tank effect significant. Corallite density in areas of new lateral fragment growth was significantly affected by light (LM, light: df = 2, F = 10.7, p = 0.0007) and genotype (genotype: df = 2, F = 4.0, p = 0.035), and there was a significant interaction between the two (light*genotype: df = 4, F = 4.2, p = 0.013), tank was also significant (tank: df = 1, F = 6.7, p = 0.018). Corallites were 50% more dense in areas of new lateral growth in the darkest light treatment (mean = 14.44 cm^2 −1^ ± 0.64) than in the full sun treatment (mean = 8.87 cm^2 −1^ ± 0.88) (Fig 3b). Although all colonies showed the same density trend from full sun to 90% treatment, the reaction norms of individual genotypes differed (Fig S3d). For Genotype 1 the full sun and 75% shade treatments were visually similar, while for Genotypes 2 and 3 the 75% and 90% shade treatments were visually similar (Fig S4).

### Coral condition metrics

**Fig 4:**
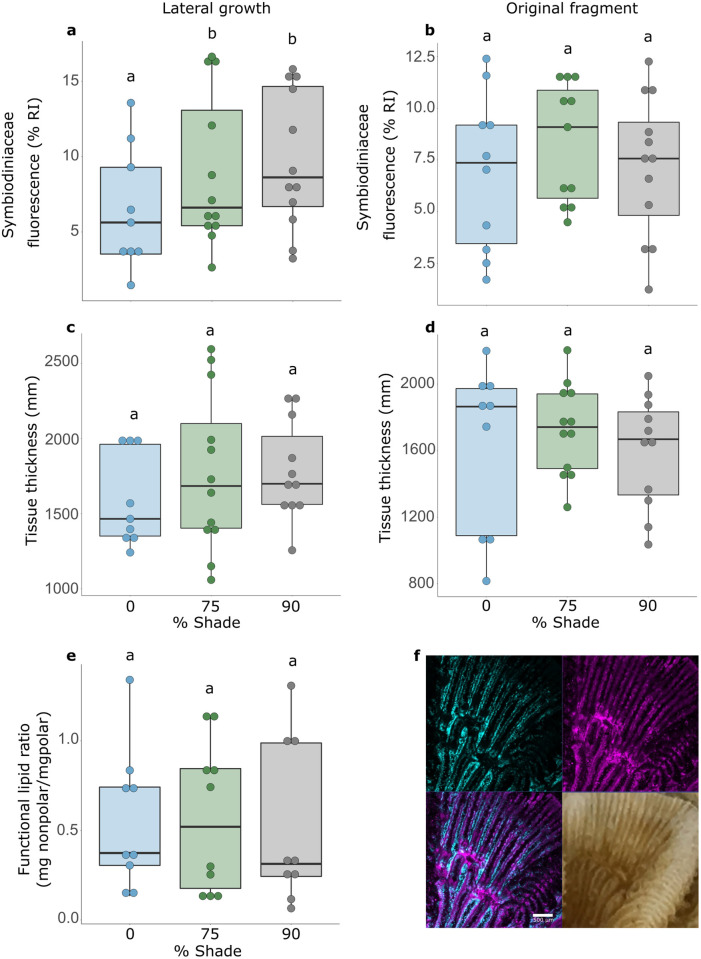
Coral conditiom metrics of *Pavona varians* across three light treatments: Symbiodiniaceae fluorescence (a,b) and tissue thickness (c,d) in the lateral growth **(a,c), and original fragments (b,d) of *Pavona varians* across three light treatments.** Functional lipid ratio of the whole fragment **(e)**. Photograph and Laser Scanning Confocal Microscopy image of P. varians fragment **(f)**. Cyan, coral tissue fluorescence; magenta, Symbiodiniaceae fluorescence. Scale bar, 0.5 mm. Letters correspond to significant pairwise differences in post hoc analysis (p < 0.05).

In areas of new lateral growth, mean tissue thickness was significantly affected by genotype (genotype: df = 2, F = 36.12, p < 0.0001), but not light or tank. Mean Symbiodiniaceae fluorescence in areas of new lateral growth was significantly affected by light (LM: df = 2, F = 6.67, p = 0.0052) and genotype (df = 2, F = 60.12, p < 0.0001). Fluorescence attributed to Symbiodiniaceae was approximately twice as high in the darkest treatment (mean = 9.79 RI 100% ± 0.602) compared to under 0% shade (mean = 5.59 RI 100% ± 0.728) (Fig 4a).

In contrast to areas of new lateral fragment growth, light did not affect tissue thickness or Symbiodiniaceae fluorescence in the original fragment. There was no significant effect of light on tissue thickness of the original fragment, but there was a significant effect of genotype (LM, genotype: df = 2, F = 46.32, p = < 0.0001), and a significant interaction (light*genotype: df = 4, F = 3.5, p = 0.023). Similarly, there was no significant effect of light on fluorescence in the original fragment, but genotype was significant (LM, genotype: df = 2, F = 25.89, p = < 0.0001).

For the entire fragments, total lipids were not significantly different among light treatments or genotypes. The ratio of nonpolar to polar lipids was also not significantly different among light treatments but there was a significant genotype effect (LM, genotype: df = 2, F = 4.8, p = 0.02) (Fig 4e).

### Symbiodiniaceae community

**Fig 5:**
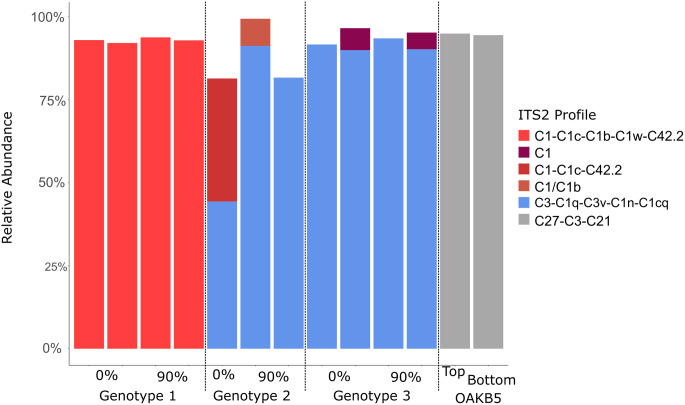
Relative abundance of ITS2 profiles for each genotype of *P. varians* included in analysis, from 0% and 90% shade, and from two samples of OAKB5, profiles generated in SymPortal [74]. Each bar represents a fragment from different light treatments of the same genotype.

All subsamples from experimental and field fragments contained symbiont communities dominated by the genus *Cladocopium*, but phylotype varied between genotypes. Genotype 1 communities predominantly contained C1 symbionts, with no variation across light treatment. Genotypes 2 and 3 were dominated by C3, with the exception of the full-sunlight fragment of Genotype 3 which has a mixture of C1 and C3. Finally, the OAKB5 (Fig 1) symbiont community was different from experimental corals, dominated by C27, but again community composition did not vary across colony surfaces exposed to different light levels, i.e., top and bottom of the colony (Fig 5). There is a single sample point from Genotype 2 in the 0% shade treatment as the other sample’s sequencing run failed.

### Model of additive effects of light and temperature

**Fig 6:**
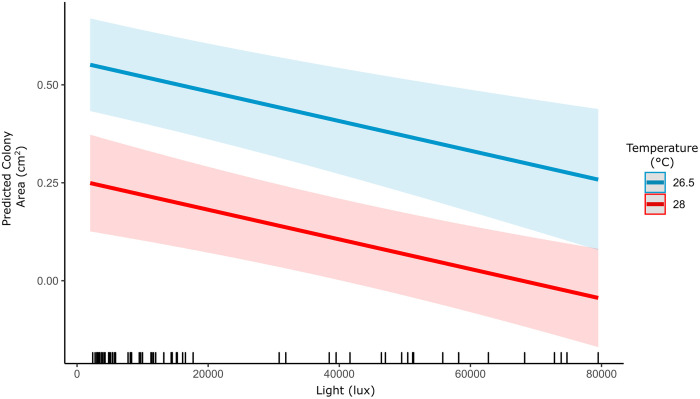
Predictions for lateral growth under normal (26.5 °C) and bleaching temperatures (28 °C) across light, from Linear Mixed Effects Model, shading indicates 95% confidence interval of the effects of light and temperature. Rug plot indicates distribution of experimental data.

The best linear mixed effects model included the effects of temperature, light, and genotype but not interactions between these predictors. The next best model included an interaction between light and genotype, but support for this model was weak (△AICc 5.31). Therefore, the best-fitting model showed that light and temperature each had negative, additive (and independent) effects on growth (Fig 6). Further, cross-validation between the best model LMM and the Generalized Additive Model showed these effects were linear, with no significant difference in the mean squared predictive error (<1%), and otherwise similar error distributions between the two models. Under the LMM, lateral growth was reduced by both increasing temperature (LMM: Estimate = −0.1092 cm^2^ ± 0.024, t = −4.567, p < 0.0001) and increasing light (LMM: Estimate = −0.09047 cm^2^ ± 0.026, t = −3.997, p = 0.0002), and both had similar effect sizes (△AICc 8.68). Lateral growth was consistently 0.3 cm less at 28°C than at 26.5°C, and this difference was offset by the 0.29 cm increase in growth in the darkest to brightest light recorded (2,000–80,000 lux).

## Discussion

The common garden experiment, symbiont metabarcoding, and growth rate modeling revealed four main findings. First, *P. varians* is phenotypically plastic in response to light, changing its morphology in ways that are consistent with an adaptive response. Second, growth rates of *P. varians* were highest under the darkest experimental conditions. Third, in contrast to the plasticity of the host’s morphology, Symbiodiniaceae community was not affected by light; however, symbiont community composition varied between genotypes. Finally, light and temperature had equal and additive negative effects on growth, indicating that low light can partially mitigate the negative effects of high temperatures on growth. Taken together, these findings indicate that phenotypic plasticity may be a key mechanism by which *P. varians* achieves a broad realized niche; either by persisting in place through changing conditions, or by allowing range shifts at the scale of the species after larval dispersal of individuals to newly vacant habitats or use of refuges.

### Morphological plasticity

The growth experiments suggest that much of the inter- and intracolony morphological variation observed in *P. varians* on natural reefs is due to phenotypic plasticity in response to light. In high light, experimental fragments grew more slowly (by either mass or 2D footprint) but had more rugose skeletons (taller and more ridges), and a lower density of corallites (Figs 2,3, S4,5 Figs). In low light, fragments grew more quickly and had flatter skeletons with shorter ridges, and a higher density of corallites. Although we used only three genotypes in this experiment, due to practical constraints (primarily permits for coral sampling) and removal of a fourth colony after the experiment because genetic screening revealed it to be a different, cryptic lineage; our findings were still statistically well-supported. Further investigation with more genotypes might shed light on the extent of genetic variation in the presence and extent of the plastic response within the population of *P. varians* in Hawaiʻi. Similarly, field experiments comparing rugostiy of multiple colonies under different light regimes would be an interesting supporting natural experiment.

This morphological phenotypic plasticity of rugosity and corallite density could be adaptive, because it may allow individual colonies to improve their performance under different light conditions. First, under high light, increased ridge formation potentially enhances self-shading of zooxanthellae (in the furrows between ridges) from excess light, while in low light conditions, flatter skeletons can increase light capture by zooxanthellae [79–83] Second, plasticity in corallite density may be an integrated phenotypic response to variation in light availability because greater corallite (i.e., polyp) densities under low light conditions may increase the potential for colonies to offset reductions in photosynthesis by feeding on zooplankton [84], but see [85]. This contrasts with other studies assessing light-associated plasticity of corallite density and morphology which generally found lower corallite density and larger corallites with increasing depth [86–88], our results are more similar to results of morphological variation for the depth generalist species *Monastrea cavernosa* [89] and *Pocillpora damicornis* [87]. Mixotrophy has not been established for *P. varians*; however several other species of *Pavona* and *Leptoseris* are mixotrophic, with greater reliance on heterotrophy at depths from 70 to 120 m [54, 90]. Traits related to increased resource acquisition, such as branching frequency and rhizome formation, are similarly plastic in plants, maximizing resource capture in heterogeneous light environments [91].

Skeletal plasticity in *P. varians* could provide other advantages under different light environments. Increased colony rugosity may increase movement of metabolites away from, and carbon dioxide towards, the colony surface due to increased shear stress from turbulent flow generated by the skeletal ridges [92, 93]. Similarly, colonies with irregular surfaces transfer more heat from water flow, potentially beneficial in warm, shallow water [94]. Greater corallite density in corals may also have other advantages, as more polyps may enhance autotrophy because more space is devoted to gastrovascular cavities, which in turn can accommodate larger numbers of photosynthesizing symbionts (see Fig 4f).

Genotypic variation was also significant for all metrics, but the overall trends in response to light were consistent across genotypes (S4 Fig). The effect of light on morphological traits was notably similar, despite the differences in acclimation time in the nursery prior to the experiment between some of the genotypes (G1 vs G2&3).This suggests any impact of pre-experiment acclimation time on our results was minimal.

In contrast, genotype-by-environment interactions were uncommon, indicating less genetic variation in plasticity itself. Significant underlying genetic variation affecting morphological traits is not uncommon in corals and is consistent with other studies on light-associated phenotypic plasticity [e.g., 95, 96, 97]. Our results also agree with the prevalence of individual effects in corals for other traits, such as stress tolerance [98, 99]. High underlying genetic diversity, clearly evident in a modest sampling of genotypes, could have ramifications for the response of *P. varians* to climate change, as greater standing genetic variation can lead to rapid adaptation [100, 101]. Genotypic effects may also be important for coral restoration, where targeted propagation efforts on individuals known to be hardier or faster growing (such as Genotype 1, in our study) may be the best use of scarce resources [102]. And, although all three genotypes were collected in shallow water, phylogenetic analysis of *P. varians* revealed that these genotypes were genetically indistinguishable from individuals present on deeper reefs of up to 73 m (S1 Fig), albeit using a single mitochondrial marker which may not be able to distinguish between cryptic species [103]. For deep reef refugia to be successful the shallow and mesophotic populations must be genetically connected such that barriers between these two environment do not restrict movement of gametes or larvae [104]. Beyond our genetic analysis of a handful of colonies from different depths, the degree of connectivity between shallow and deep populations of *P. varians* remains unknown, but in studies of agaricids in the Caribbean different speceis were found to be connected across both depth and islands [105]. All colonies investigated in this experiment were collected from shallow water, whether an equivalent plastic response is recorded in colonies from deeper, lower light depths is an important open question.

Although the growth rate of *P. varians* was greatest at the lowest light levels in our experiment, fragments had positive growth in each treatment, highlighting the broad light niche of *P. varians*. Few coral species can persist across such a broad depth gradient, and the most abundant species (from [41] *Porites rus, Goniastrea pectinata,* and *Echinopora lamellosa*) are all, like *P. varians*, plastic in morphology or physiology with respect to light [Jaubert 1977 in 82, 96, 106]. Light-driven plasticity may also allow *P. varians* to exploit other dark environments, such as turbid reefs, and shallow, cryptic, shaded microhabitats that could similarly serve as potential refuges for corals whose effectiveness and ecological relevance remain debated [104]. The increase in coral growth with decreasing light contrasts with general expectations for coral physiology [34, 107, 108], though trends in variation of growth across depth gradients can be species-specific [109].

### Plasticity of functional traits

Light is an important variable affecting coral growth. Light attenuates exponentially with depth, and is the main factor delineating the mesophotic zone [110, 111]. In low light, we found new lateral growth of experimental fragments had thicker tissues and higher symbiont fluorescence (Fig 4). In contrast, functional lipid ratio (FLR) did not change across light conditions (Fig 4), nor did Symbiodiniaceae community (Fig 5).

Although *P. varians* maintained positive growth under a wide range of light, plasticity in other important traits such as functional lipid ratio, the ratio of structural to storage lipids, may provide an additional and more nuanced views of how light impacted coral health [112, 70]. In plants, altered lipid concentrations can be indicative of stressful temperatures [113, 114] and drought [115]. During times of stress for corals, such as bleaching, the relative amount of non-polar storage lipids can similarly decline, as energy reserves are depleted to account for the nutritional shortfall [112, 116]. However, storage lipids may also decline when used to either fuel rapid growth [117, 66] or to offset energy demands under less photoefficient conditions [71]. The overall lack of a relationship between FLR and light gradient in our data might reflect the inability to separate similar changes in FLR in low light due to rapid coral growth, from similar changes in high light due to reduced photosynthesis. Alternatively, the absence of a relationship between FLR and light may indicate that *P. varians* can maintain FLR under a range of conditions that, in other species, drive substantial changes in energy use and storage [112, 68, 71]. FLR may also provide a measure of genotype vigor in *P. varians*, because storage lipid was consistently low in Genotype 3 under our experimental light conditions, which also bleached and had the slowest growth rate of all the colonies (S3,4ab Fig).

Symbiodiniaceae fluorescence and tissue thickness also both decline during bleaching, as symbionts are compromised and coral condition deteriorates [55]. In our experiment, the response of these metrics depended on the part of the fragment analyzed. In portions of the fragment corresponding to the original fragment, tissue thickness and fluorescence did not differ among light treatments; however, symbiont fluorescence appeared to exhibit plasticity in areas of new lateral growth in *P. varians*. Areas of new lateral growth had reduced symbiont fluorescence in full sun compared to the 90% shade treatment. In contrast, the original regions of the full-sun fragments had a more rugose skeleton (S6 Fig) with more pronounced ridges. Increased rugosity may have protected symbionts from light stress in full sun via self-shading, and prevented a measurable decline in symbiont density and tissue thickness in the original fragment. In new lateral growth in *P. varians*, the skeleton was thinner and ridges are less developed, perhaps this offered little protection from the stressful high light experienced in the full sun treatments, subsequently symbiont fluorescence declined. Further, in darker conditions higher symbiont fluorescence may facilitate greater capacity for photosynthesis when light is otherwise limited.

Although we recovered three different symbiont communities among our four colonies (dominated by C3, C1, and C27, similar to those in *Pavona* elsewhere, see [118–120]), these symbiont communities were consistent across light treatments at the end of the experiment (Fig 5). No symbiont differences between light treatments could reflect insufficient time for community shifting/sorting over the course of the 6-month experiment. However, the consistency across samples collected from the colony *in situ* (OAKB5) suggest that in the field, within a colony, symbiont community may also persist in different light environments. OAKB5 is an interesting case study because the two parts of the colony that we sampled were from what we assumed were persistent different light conditions (top and underside of a shallow ledge), and yet the Symbiodiniaceae community was consistent. High symbiont community diversity among colonies in our analysis is consistent with previous findings that *P. varians* is highly labile in its associations with Symbiodiniaceae [121], yet our results suggest *P. varians* does not rapidly shuffle symbionts in response to light availability. The relative performance of Symbiodiniaceae phylotypes is hard to determine, but C3 appears more stress tolerant than C1 [122]. In our experiment, the composition of the symbiont community was not related to the low functional lipid ratio signal of Genotype 3 (S4i Fig), as Genotype 2 and 3 were dominated by the same Symbiodiniaceae phylotype.

### Light and temperature stress

Understanding the impacts of light and temperature and other stressors in a multifactorial framework is important for predicting the future spatial distributions of corals and for management initiatives [123, 124]. Although hermatypic taxa are generally restricted to clear, tropical water, the response of photosynthetic scleractinians to climate change depends on their tolerance to both light and temperature, as the negative effects of both high light and temperature are evident from many experiments [22, 125–129]. Further, the severity of bleaching is often reduced by low light conditions, such as cloud cover [28, 29, 130], turbidity [30–33], and in shaded microhabitats such as crevices and overhangs [131]. Indeed, deep reefs are often not a temperature refuge ([35] and references therein), yet bleaching is often reduced at depth [24–27]. Indeed, a recent experimental study found thermal tolerance was maintained in two common species at mesophotic light levels, even after prolonged exposure to cooler water prior to the heat stress, suggesting that the lower light levels of mesophotic depths may be providing protection from thermal stress [132].

The results from our model are consistent with the idea that these reductions in bleaching could be the product of primarily reduced light stress at depth,see [35]. The model demonstrated that both light and temperature had linear, negative effects on growth, each with similar effect sizes but with no interaction or evidence of a synergistic effect. Thus, for *P. varians*, low light may mitigate the negative consequences of high temperatures on coral growth, without impacting growth rates as in other species [133]. This could be relevant at two scales for this species: within the colony, and across depth. First, by supporting the proposed adaptive role of increased ridge formation in high light conditions shading zooxanthellae, and thereby reducing the negative effects of increased irradiance. And second, by highlighting the importance of low light environments (such as deeper depths or overhangs) as refuges for *P. varians* [134]. The lowest light treatment never became limiting for *P. varians* in our experiment, which contrasts with other species impacted by less modest light reductions [135, 133]. We found that *P. varians* continued to grow at Hawaiʻi bleaching temperatures (at or above 28°C as defined by NOAA) in 90% shade (equivalent to ~ 50 m depth), whereas growth was halted in full sunlight (~0.5 m depth) at the same temperature. Although we did not monitor light in the most functionally relevant unit (PAR), the HOBO loggers demonstrate an order-of-magnitude variation in light in this experiment. This difference in light encompasses the full spectrum of irradiance that can sustain shallow-water, coral reef accretion [136]. Lastly, flow, especially at moderate rates, can reduce the harmful effects of temperature in experimental settings and should be considered when discussing bleaching and recovery in the complex conditions of natural reefs [137–139].

The shading treatments used in this study did not replicate the light conditions of mesophotic depths, which have reduced irradiance, are spectrally different compared to shallow depths, and have a narrower light field [107]. Light wavelengths are attenuated unevenly when descending through the water column: red-yellow are lost rapidly while blue-UV continues to penetrate to deeper depths [140]. The biological consequences of this spectral loss to the corals found at mesophotic depths are thought to be largely constrained by the limitations of the photosynthetic apparatus of Symbiodiniaceae which cannot readily change their photopigments or photosystems [107], although *Stylophora pistillata* has shown adaptation to blue light [141]. Other studies have recovered chlorophyll ratio changes in corals which do not fit theoretical expectations [107, 142, 143].

### Generalists in a warming world

Anthropogenic climate change is causing shifts in community composition of both terrestrial and marine biota [144–149]. While exact responses differ between ecosystems, historical extinction events and niche theory predict that generalists are more likely than specialists to persist through the current human-induced stress event [150–152]. Shifts from specialists to generalists and functional homogenization in response to current climate change are already evident on land and in the sea [153, 154].

Phenotypic plasticity plays an important role in shifts to higher altitudes observed in many mountain plant species in response to warming temperatures at lower elevations [155, 156]. The DRRH offers a similar mechanism through which corals could persist through periods of heat stress. In the marine environment, deeper depths correspond to not only lower temperatures but also declines in light, an essential resource affecting coral growth. Therefore light and depth generalist coral species may be able to use these as temperature refugia, and be disproportionately important as early colonists on future reefs.

Our study focused on a “deep generalist” species present on reefs across a wide range of depths but which is more abundant on deep reefs [41]. Although the extent to which the abundance of corals is limited by maximum growth rate or environmental constraints on growth (i.e., competition and disturbance) has been rarely tested [40], the outcome of our experiment suggests that growth rate reaction norms of *P. varians* may be a contributing factor to the depth-related abundance of this species. If restricted to deep reef refuges, deep generalists like *P. varians* may disproportionately contribute to future shallow water recruitment if faster growth and therefore larger colony sizes still translates into greater larval production at depth like it does in shallower water [157,158]. But, potential increased relative abundance on shallow reefs may be diminished by intrinsically slower growth in high light. Comparative studies that relate growth rates under controlled conditions to field studies of realized growth on reefs for both deep and shallow generalists will be necessary to identify taxa most likely to benefit from the DRRH.

## Supporting information

S1 Fig Bayesian phylogeny of Cox1–1-rRNA intron sequences from Agaricia, Leptoseris, and Pavona. **Includes** 18 published samples (DQ*,KF*, KR*:Medina et al. 2006; Luck et al. 2013; Waheed et al. 2015), the three genotypes collected for this study and OAKB5 (in bold). Tip labels indicate site, depth collected (m) where available, and sample code or genbank accession number in parentheses. *P. varians* samples ordered by increasing depth of collection.
(TIF)

S2 Fig Experimental Set Up. A. Schematic of tank set-up for the experiment. B. Photograph of the two tanks and treatments. Red arrow indicates one of the experimental tiles on the suspended tray. The red numbers indicate different light treatments: 0, 2, 4x layers of shade cloth in each treatment.
(TIF)

S3 Fig Experimental Conditions. Average light and temperature through time, in three light treatments. And condition of coral colonies through time, as measured percent of total coral surface area. Each set of 3 bars corresponds to the light treatments.
(TIF)

S4 Fig Genotype x Environment Interactions. Genotype by a) lateral growth rate * b) calcification rate, c) rugosity, d) corallite density of lateral growth*, e) Symbiodiniaceae fluorescence in original fragment, f) tissue thickness in original fragment* g) Symbiodiniaceae fluorescence in lateral growth, h) tissue thickness in lateral growth, i) functional lipid ratio. Asterisk signifies traits with significant gxe interaction.
(TIF)

S5 Fig Principal Components Analysis of measured traits in *P. varians* across three light treatments. PCA of dimensions 1 & 2, collectively 70% of variation, arrow length corresponds to contribution of each variable to dimensions.
(TIF)

S6 Fig Rugosity of the whole colony and the original fragment. All measurements taken at the end of the experiment.
(TIF)

S1 Text Supplemental Methods, Results, and References. Additional information.
(DOCX)

S1 Data Intron sequences. Cox1–1-rRNA intron sequences for three experimental genotypes and OAKB5.
(CSV)

S2 Data End-Point Data. End-point data from the 6-month light experiment.
(CSV)

S3 Data Environmental Data. Temperature, light, and growth data used for linear modeling.
(CSV)

S4 Data ITS2 Profile Keys. Key to composition of ITS2 profiles in the ITS2 abundance plots.
(CSV)

S5 Data ITS2 Data Abundances. Abundances of each profile of ITS2 in each coral sample.
(CSV)
