# Supplementary figures and images for: Light-driven phenotypic plasticity in the depth-generalist coral, Pavona varians
Source: PLoS One. 2025 Jul 1;20(7):e0326069. doi: 10.1371/journal.pone.0326069 (PMC12212529; doi:10.1371/journal.pone.0326069)

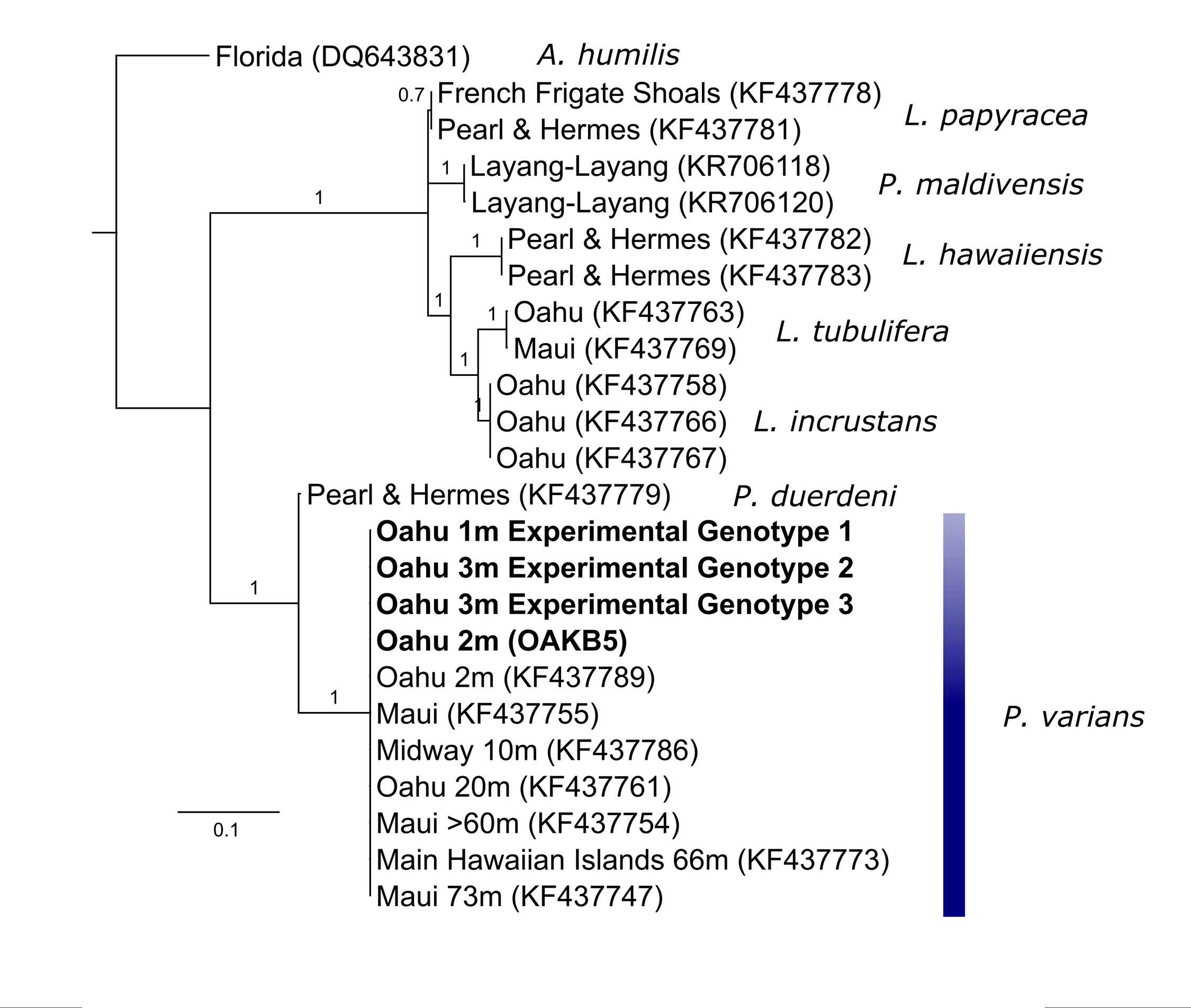

Supplement: S1 Fig — Includes 18 published samples (DQ*,KF*, KR*:Medina et al. 2006; Luck et al. 2013; Waheed et al. 2015), the three genotypes collected for this study and OAKB5 (in bold). Tip labels indicate site, depth collected (m) where available, and sample code or genbank accession number in parentheses. P. varians samples ordered by increasing depth of collection. (TIF) [file pone.0326069.s001.tif]

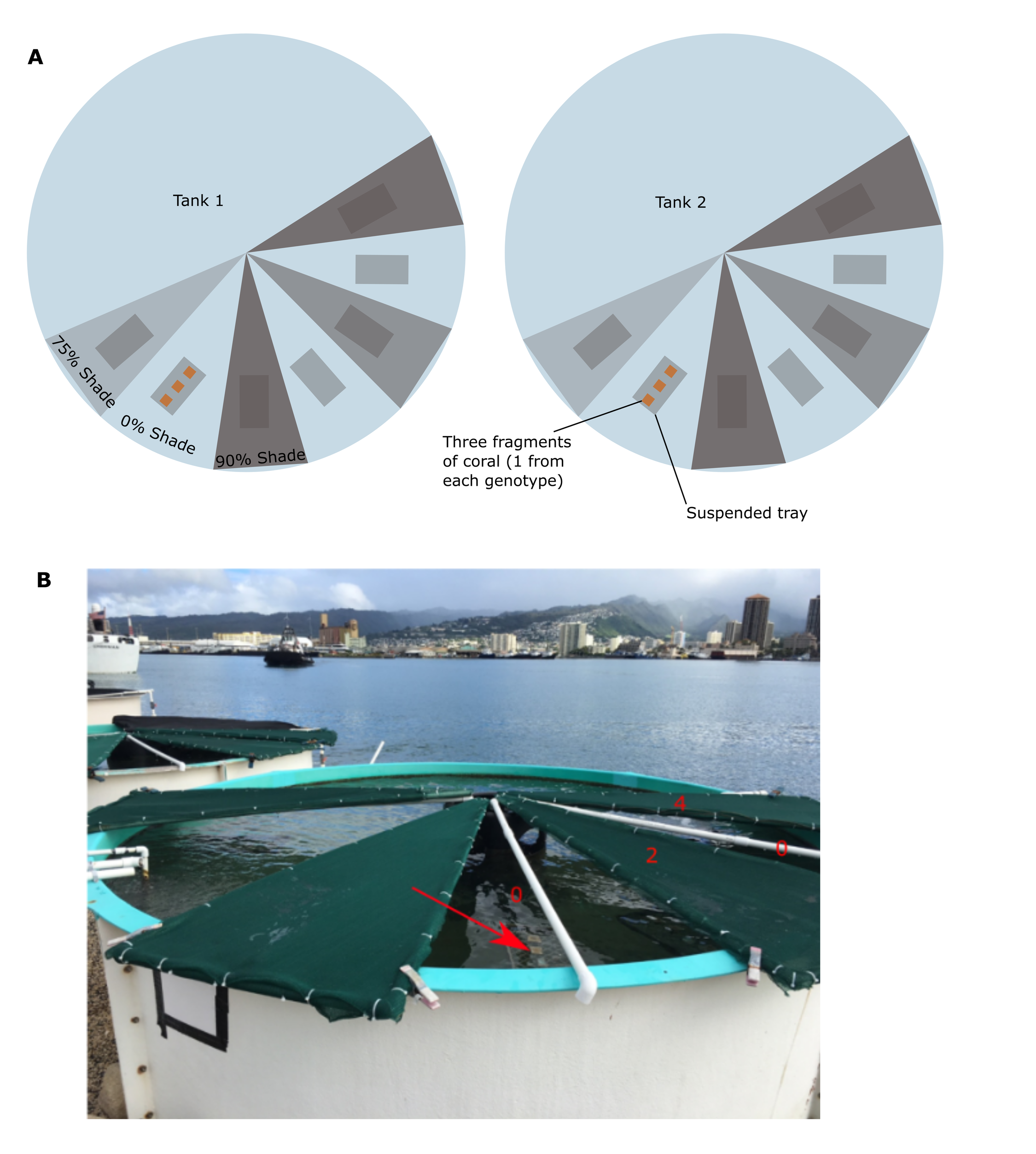

Supplement: S2 Fig — Schematic of tank set-up for the experiment. B. Photograph of the two tanks and treatments. Red arrow indicates one of the experimental tiles on the suspended tray. The red numbers indicate different light treatments: 0, 2, 4x layers of shade cloth in each treatment. (TIF) [file pone.0326069.s002.tif]

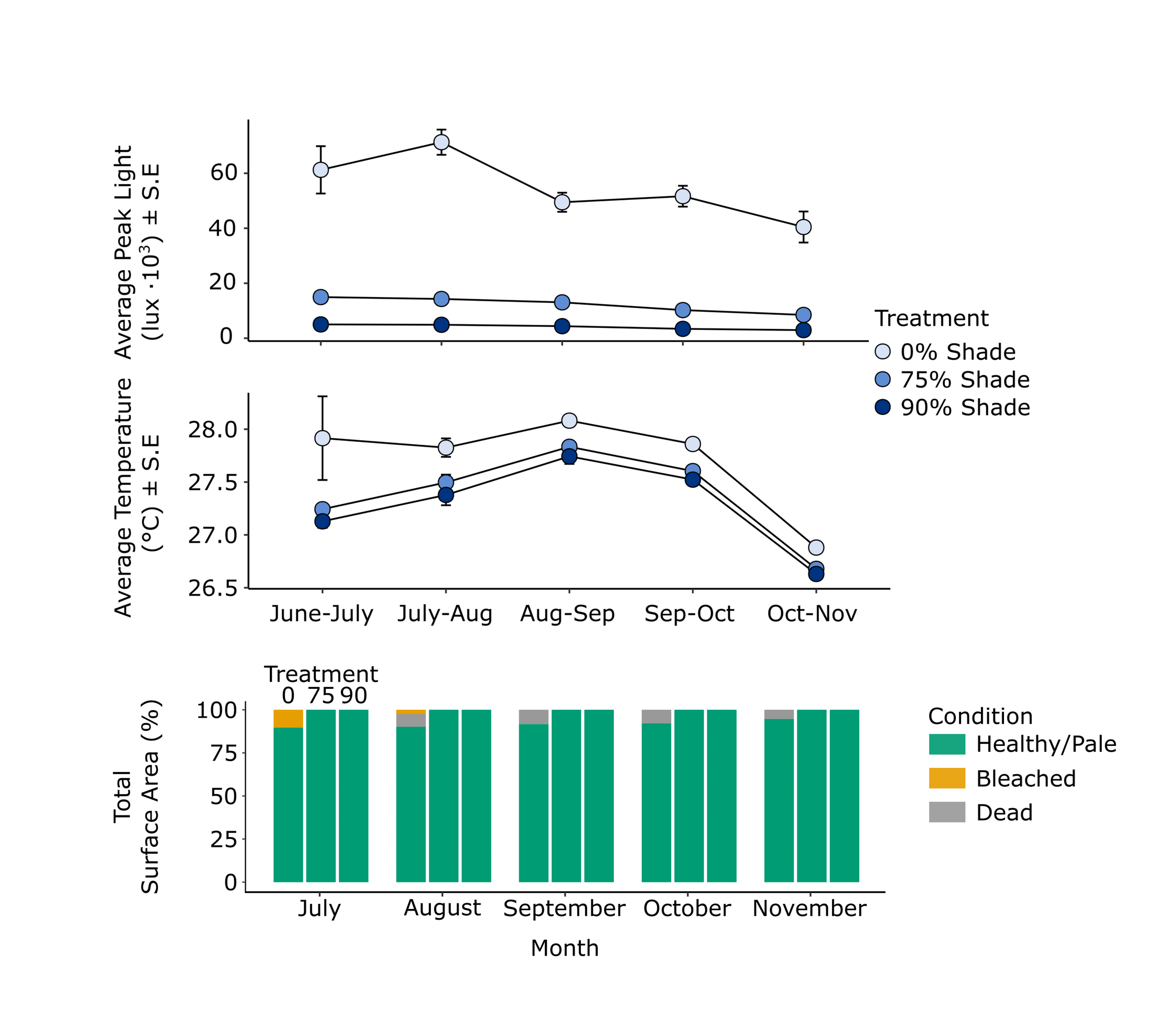

Supplement: S3 Fig — Average light and temperature through time, in three light treatments. And condition of coral colonies through time, as measured percent of total coral surface area. Each set of 3 bars corresponds to the light treatments. (TIF) [file pone.0326069.s003.tif]

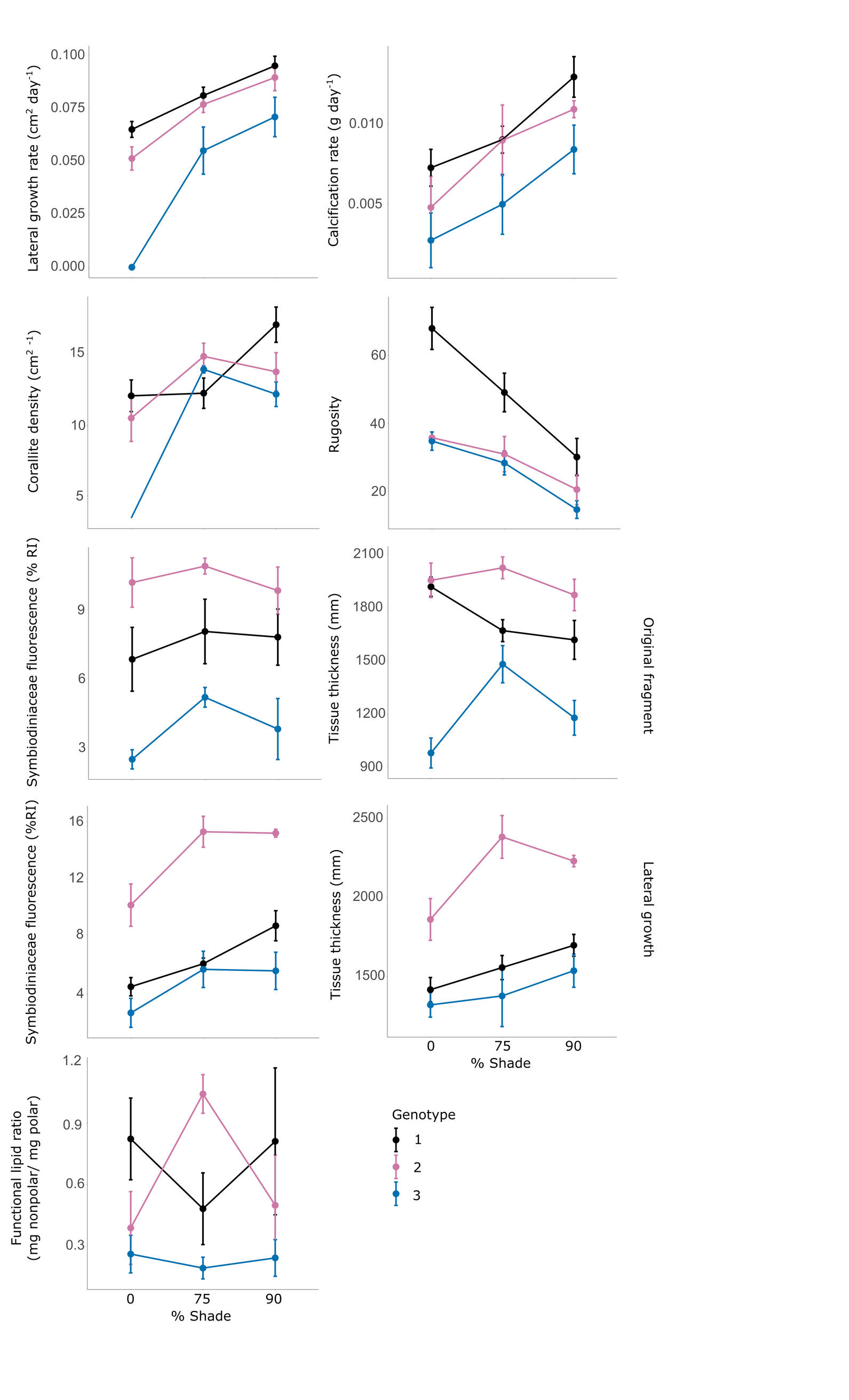

Supplement: S4 Fig — Genotype by a) lateral growth rate * b) calcification rate, c) rugosity, d) corallite density of lateral growth*, e) Symbiodiniaceae fluorescence in original fragment, f) tissue thickness in original fragment* g) Symbiodiniaceae fluorescence in lateral growth, h) tissue thickness in lateral growth, i) functional lipid ratio. Asterisk signifies traits with significant gxe interaction. (TIF) [file pone.0326069.s004.tif]

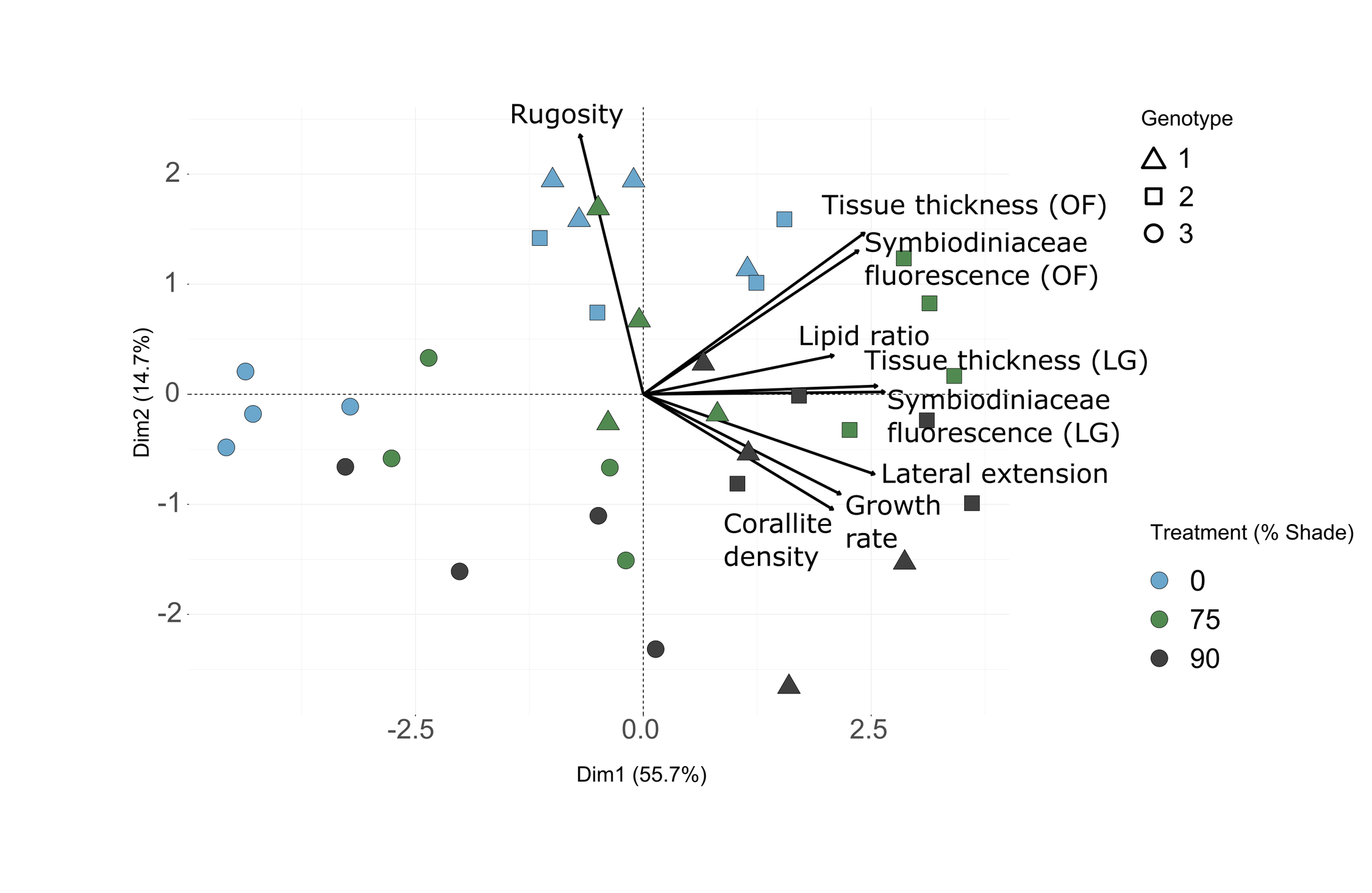

Supplement: S5 Fig — PCA of dimensions 1 & 2, collectively 70% of variation, arrow length corresponds to contribution of each variable to dimensions. (TIF) [file pone.0326069.s005.tif]

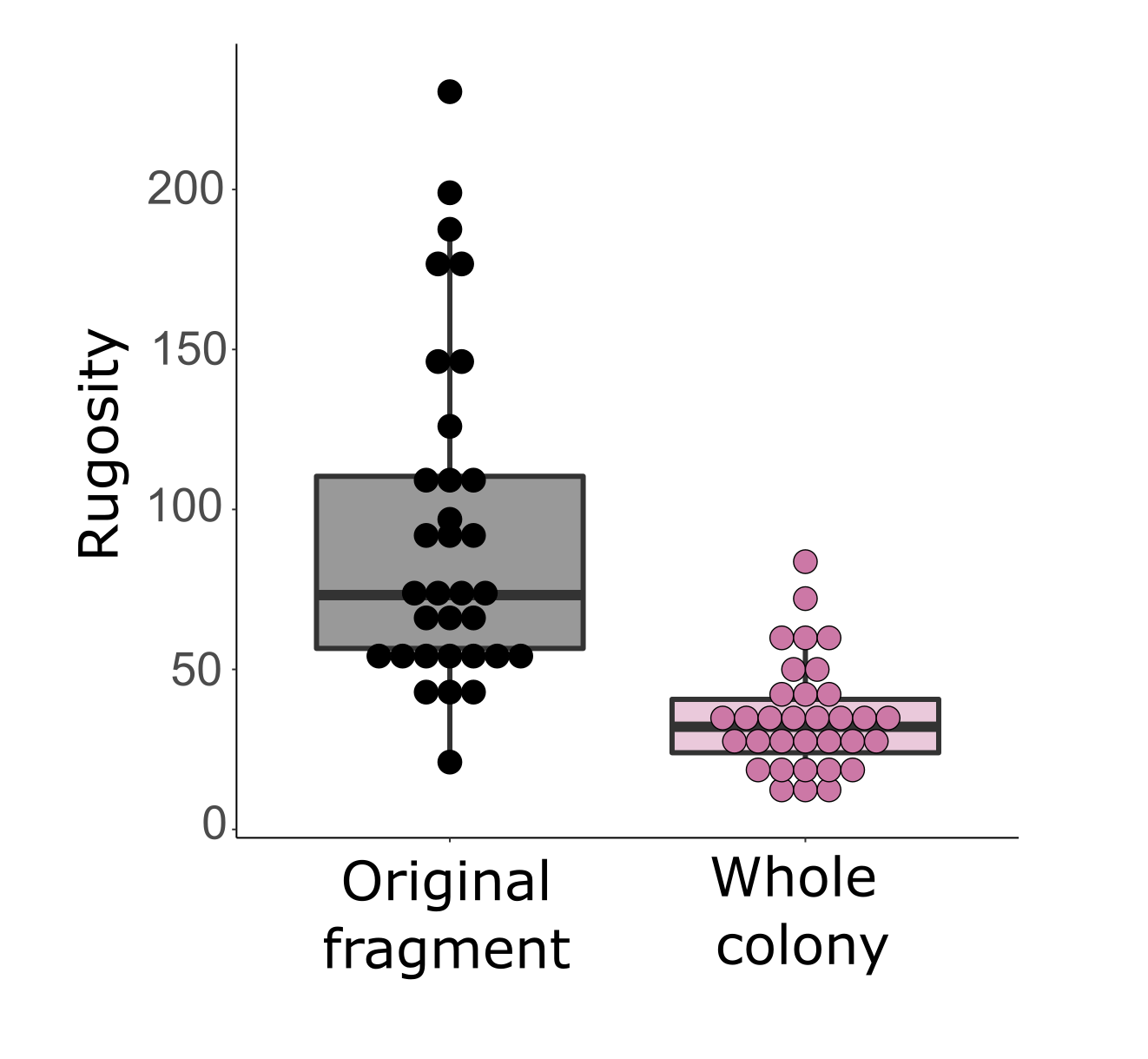

Supplement: S6 Fig — Rugosity of the whole colony and the original fragment. All measurements taken at the end of the experiment. (TIF) [file pone.0326069.s006.tif]
