# Supplementary material for: Light-driven phenotypic plasticity in the depth-generalist coral, Pavona varians
Source: PLoS One. 2025 Jul 1;20(7):e0326069. doi: 10.1371/journal.pone.0326069 (PMC12212529; doi:10.1371/journal.pone.0326069)
Supplement: S1 Text — Supplemental Methods, Results, and References. Additional information. (DOCX) [file pone.0326069.s007.docx]

**SUPPLEMENTARY MATERIALS**

**Methods**

**Molecular Analyses**

To assess whether individuals of *P. varians* collected for this experiment form a single clade, 663-670 bp of the *cox*1-1-rRNA intron (Luck et al. 2013) was sequenced from the experimental colonies. We also sequenced a fragment of one other colony exhibiting high intracolony morphological variation from Kaneohe Bay (OAKB5), collected in 2016 (Fig. 1). The mitochondrial intron is variable enough to distinguish species within Agariciidae, and is consistent with morphological species (Luck et al. 2013, Waheed et al. 2015; Terraneo et al. 2017). DNA was extracted using a Qiagen DNeasy kit according to the manufacturer’s recommendations. The intron was amplified from six colonies using primers and protocol described in Luck et al. (2013). PCR products were sequenced using an Applied Biosystems 3730XL DNA Analyzer at the University of Hawaiʻi at Mānoa’s Advanced Studies in Genomics, Proteomics and Bioinformatics (ASGPB) sequencing facility.

Sanger sequences were manually checked and edited using Geneious R10 and aligned with other electronically-retrieved agaricids (Medina et al. 2006; Luck et al. 2013; Waheed et al. 2015) using ClustalW in Geneious with default parameters. A phylogeny was built using the Bayesian inference criterion implemented in the MrBayes 3.2.6 (Huelsenbeck and Ronquist 2001) Geneious R10 plug-in, under the HKY85 + G model, chosen with ModelFinder and BIC (Trifinopoulos et al. 2016; Kalyaanamoorthy et al. 2017). The phylogenetic analysis ran for 2,500,000 generations on four Markov chains, sampling every 1,000 generations. The first 100,000 generations were discarded as burn-in after examining the trace for convergence.

**PCA**

To visualize trends in the different metrics, a Principal Component Analysis (PCA) was conducted using nine variables: growth rate, calcification rate, corallite density, rugosity, functional lipid ratio, tissue thickness (in the original fragment, and lateral growth), and Symbiodiniacaea fluorescence (in the original fragment, and lateral growth). To generate a full data matrix for PCA, missing data (34/324 cells) were imputed using MissMDA (v1.14, Josse and Husson 2016). PCA was created using FactoMineR (v1.42, Lê et al. 2008), and visualized using the factoextra package (v0.5-3.0) in R.

**Results**

**Phylogeny**

Analysis of *cox*-1-rRNA intron sequences showed all the experimental colonies, plus OAKB5, formed a clade with six colonies identified by Luck et al. (2013) as *P. varians*; and these sequences are > 99.5% identical (Fig. S1). This clade included electronically-retrieved sequences from samples collected from shallow (2 m) to mesophotic depths (73 m). *P. varians* was placed in the tree as sister to *P. duerdeni*, a massive coral, highly morphologically distinct from *P. varians*.

**Genotype Response Temperature and Light**

Other variables also had a significant effect on growth, consistent with the rest of the experiment. Genotype had a significant effect on growth, with Genotype 1 having a significant positive effect on growth (LMM: Estimate=0.5746 cm^2^ ± 0.084, t =-6.88, p < 0.0001), whereas Genotype 3 had a significant negative effect on growth (LMM: Estimate=-0.2448 cm^2^ ± 0.048, t =-5.132, p < 0.0001). Unexpectedly, the area of the colony at the previous time point also had a negative effect on growth (LMM: Estimate=-0.05890cm^2^ ± 0.048, t =-5.132, p < 0.0001).

**Works Cited**

Huelsenbeck, J. P., & Ronquist, F. (2001). MRBAYES: Bayesian inference of phylogenetic trees. *Bioinformatics* , *17*(8), 754–755.

Josse, J., & Husson, F. (2016). MissMDA: A package for handling missing values in multivariate data analysis. Journal of Statistical Software, 70(1).<https://doi.org/10.18637/jss.v070.i01>

Kalyaanamoorthy, S., Minh, B. Q., Wong, T. K. F., von Haeseler, A., & Jermiin, L. S. (2017). ModelFinder: fast model selection for accurate phylogenetic estimates. *Nature Methods*, *14*(6), 587–589.

Lê, S., Josse, J., Husson, F., & Others. (2008). FactoMineR: an R package for multivariate analysis. Journal of Statistical Software, 25(1), 1–18.

Medina, M., Collins, A. G., Takaoka, T. L., Kuehl, J. V., & Boore, J. L. (2006) Naked corals: Skeleton loss in Scleractinia. *Proceedings of the National Academy of Sciences of the United States of America, 103*(24), 9096–9100.

Terraneo, T. I., Arrigoni, R., Benzoni, F., Tietbohl, M. D., & Berumen, M. L. (2017). Exploring the genetic diversity of shallow-water Agariciidae (Cnidaria: Anthozoa) from the Saudi Arabian Red Sea. *Marine Biodiversity,* *47*(4), 1065–1078.

Trifinopoulos, J., Nguyen, L.-T., von Haeseler, A., & Minh, B. Q. (2016). W-IQ-TREE: a fast online phylogenetic tool for maximum likelihood analysis. *Nucleic Acids Research*, *44*(W1), W232–W235.

Waheed, Z., Benzoni, F., van der Meij, S. E. T., Terraneo, T. I., & Hoeksema, B. W. (2015). Scleractinian corals (Fungiidae, Agariciidae and Euphylliidae) of Pulau Layang-Layang, Spratly Islands, with a note on *Pavona maldivensis* (Gardiner, 1905). *ZooKeys*, *517*, 1–37.
